# 547 Clinical Outcomes for Burned Patients with Covid-19

**DOI:** 10.1093/jbcr/irac012.175

**Published:** 2022-03-23

**Authors:** Elliot Walters, Nikhil Shah, Steven E Wolf

**Affiliations:** University of Texas Medical Branch, Dickinson, Texas; University of Texas Medical Branch, Galveston, Texas; University of Texas Medical Branch at Galveston, Galveston, Texas

## Abstract

**Introduction:**

The COVID-19 epidemic has affected all aspects of medical care including a reduction in elective procedures, however, the incidence of burns and treatment for this condition has continued undaunted. Some of these patients were also diagnosed with COVID-19 infection, but it is unclear what effect, if any the SARS-CoV 2 virus has on patients recovering from a burn injury. In this study we examined the outcomes of burned patients with a concomitant diagnosis of SARS-CoV 2 virus.

**Methods:**

We examined a de-identified database of patient electronic medical records across 55 health care associations containing over 75 million patients. ICD 10 codes were used to identify those with thermal or chemical burns from January 1, 2020 to July 31, 2021 and those also diagnosed with Sars-CoV 2 virus infection within 1 month of injury. We found 49,501 patients suffered burns during the study time period; of these 474 patients (0.96%) also experienced a concomitant COVID-19 infection. We compared outcomes based on ICD 10 and CPT codes.

**Results:**

We found no significant increase in mortality between groups during the study period. However, we did find a significant increase in infections, pneumonia, respiratory failure and sepsis in those with Sars-CoV infection (p< 0.05). However, there was no significant increase in ventilator management days (p >0.05) In terms of wound healing, patients with COVID-19 also experienced significantly more excision and grafting procedures and had a higher incidence of hypertrophic scarring (p< 0.05).

**Conclusions:**

COVID-19 infection is well known to worsen respiratory outcomes, but in burned patients was also associated with an increase in other infections and poorer wound outcomes. These outcomes may emanate from a change in inflammatory status for patients with the SAR-CoV 2 virus infection. This is the first broad-based study to examine outcomes of burn victims with concomitant SARS-CoV 2 infection. Further investigation is indicated as more long-term data becomes available.

